# Influence of renal function on blood pressure control and outcome in thrombolyzed patients after acute ischemic stroke: *post-hoc* analysis of the ENCHANTED trial

**DOI:** 10.3389/fendo.2024.1341902

**Published:** 2024-12-09

**Authors:** Xinwen Ren, Chen Chen, Xia Wang, Qiang Li, Yang Zhao, Shoujiang You, Menglu Ouyang, Thompson Robinson, Richard I. Lindley, Hisatomi Arima, Xiaoying Chen, John Chalmers, Craig S. Anderson, Lili Song

**Affiliations:** ^1^ The George Institute for Global Health, Faculty of Medicine, University of New South Wales, Sydney, NSW, Australia; ^2^ Department of Stroke, The George Institute for Global Health China, Beijing, China; ^3^ Neurology Department, Shanghai East Hospital, School of Medicine, Tongji University, Shanghai, China; ^4^ Department of Neurology and Clinical Research Center of Neurological Disease, The Second Affiliated Hospital of SooChow University, Suzhou, China; ^5^ Department of Cardiovascular Sciences and National Institute for Health and Care Research (NIHR) Leicester Biomedical Research Centre, Leicester, United Kingdom; ^6^ Westmead Clinical School, University of Sydney, Sydney, NSW, Australia; ^7^ Department of Public Health, Fukuoka University, Fukuoka, Japan; ^8^ Facultad de Medicina, Clinica Alemana Universidad del Desarrollo, Santiago, Chile

**Keywords:** renal function, thrombolysis, acute ischemic stroke, clinical outcome, blood pressure

## Abstract

**Background:**

The effect of renal impairment in patients who receive intravenous thrombolysis for acute ischemic stroke (AIS) is unclear. We aimed to determine the associations of renal impairment and clinical outcomes and any modification of the effect of intensive versus guideline-recommended blood pressure (BP) control in the BP arm of the International Enhanced Control of Hypertension and Thrombolysis Stroke Study (ENCHANTED).

**Methods:**

We conducted a *post-hoc* analysis of the ENCHANTED BP arm, which involved 2,196 thrombolyzed AIS patients. Logistic regression models were used to define the association between eGFR and clinical outcomes of death, death or major disability [modified Rankin scale (mRS) scores 3–6], and major disability (mRS 3–5) at 90 days.

**Results:**

Of the 2,151 patients with available baseline renal function data (mean age 66.9 years; 38% women), 993 (46.2%), 822 (38.2%), and 336 (15.6%) had normal (eGFR ≥ 90 mL/min/1.73 m^2^), mildly (60–89), and moderate-to-severely impaired (<60) renal function, respectively. Compared with patients with normal eGFR, mortality was higher in those with moderate-to-severe renal impairment (adjusted odds ratio 1.77, 95% confidence interval 1.05–2.99; *p* = 0.031 for trend). However, the difference in death or major disability (mRS 3–6) was not significant between groups. There was no heterogeneity in the effect of intensive versus guideline-recommended BP-lowering treatment on death by grades of renal function (*p* for interaction = 0.545).

**Discussion:**

The presence of moderate-to-severe renal impairment is associated with increased mortality in thrombolyzed patients with AIS. Renal function does not modify the effect of early intensive BP-lowering treatment on death in this patient group.

## Introduction

Both stroke and chronic kidney disease (CKD) are common public health concerns that caused 143.2 million disability-adjusted life years (DALYs) and 41.5 million DALYs in 2019, respectively (1), and often occur as comorbidities (2). Renal impairment is an independent risk factor for serious cardiovascular disease, including stroke (3, 4). While studies have reported an association between renal impairment and poor functional outcome in patients with acute ischemic stroke (AIS) (5, 6), data are more limited in those who receive intravenous thrombolysis and BP-lowering treatment where the risk of intracerebral hemorrhage (ICH) and other adverse outcomes may be increased (7–10).

The blood pressure (BP) arm of the International Enhanced Control of Hypertension and Thrombolysis Stroke Study (ENCHANTED) trial showed that intensive BP-lowering treatment did not improve clinical outcomes after AIS, despite reducing the risk of intracranial hemorrhage (11). It is unknown as to whether there is an interaction between intensive BP-lowering treatment and renal function on clinical outcomes in this patient group. On the one hand, intensive BP control may cause potential renal impairment from a decrease in renal perfusion pressure and blood flow, and this may in turn lead to a higher risk of cerebral bleeding and poor functional outcome. Moreover, BP variability may be influenced by renal functional status in relation to therapeutic and psychological resistance (12). On the other hand, intensive BP-lowering treatment may have added benefits in “high-risk” patients with renal impairment who receive intravenous thrombolysis for AIS. Thus, we conducted a *post-hoc* analysis of the ENCHANTED trial to assess the prognostic significance of renal impairment and its interaction with the randomized treatment on clinical outcomes in thrombolyzed AIS patients.

## Methods

### Study design

ENCHANTED was an international, multicenter, prospective, quasifactorial, randomized, open-label blinded-endpoint assessed trial, for which the main results have been presented elsewhere (11, 13). For the BP arm, 2,196 eligible patients were randomly assigned (1:1, by means of a central web-based system) within 6 h of stroke onset to receive either intensive [target systolic BP (SBP) 130–140 mmHg within 1 h] or guideline (target SBP <180 mmHg) BP-lowering treatment over 72 h after the commencement of intravenous thrombolysis. The study protocol was approved by the ethics committees of each participating center, and written informed consent was obtained from the patients or an appropriate surrogate. Baseline demographic and clinical characteristics were obtained at the time of hospital presentation. Stroke severity was measured on both the Glasgow Coma Scale (GCS) and the National Institutes of Health Stroke Scale (NIHSS) at baseline, 24 h, and 7 days (or earlier on discharge from the hospital).

### Measures

Non-invasive BP monitoring was undertaken using an automated device applied to the non-hemiparetic arm (or right arm in the case of coma or tetraparesis), with the patient resting supine for at least 3 min in accordance with a standard protocol. Following thrombolysis, BP measurements were recorded every 15 min for 1 h, hourly from 1 to 6 h, and 6-hourly from 6 to 24 h. Thereafter, BP was recorded twice daily for 1 week (or until hospital discharge or death, if earlier). An admission blood test was conducted before the thrombolysis. Estimated glomerular filtration rate (eGFR) was calculated using the Chronic Kidney Disease–Epidemiology Collaboration equation (14). Renal function was classified into three stages: G_1_, normal (eGFR ≥ 90 mL/min/1.73 m^2^); G_2_, mild impairment (eGFR 60–89); and G_3_, moderate-to-severe impairment (eGFR < 60) (15).

Clinical outcomes were death, major disability [modified Rankin scale (mRS) scores 3–5], death or major disability (mRS scores 3–6), and physical function (ordinal shift in mRS scores) at 90 days. The key safety outcome was ICH according to centrally adjudicated brain images in association with symptoms: symptomatic intracerebral hemorrhage (sICH) was ICH associated with substantial neurological deterioration or death, according to the Safe Implementation of Thrombolysis in Stroke-Monitoring Study (SITS-MOST) definition of a large or remote parenchymal ICH (type 2, defined as >30% of the infarcted area affected by hemorrhage with mass effect or extension outside the infarct) combined with neurological deterioration (≥4 points on the NIHSS) or leading to death within 24–36 h (16). Other criteria for sICH were also used in this study.

### Statistical analysis

Demographic and baseline clinical characteristics were presented as mean with standard deviation (SD) or median with interquartile range (IQR) for continuous variables and numbers with percentages for categorical variables. Baseline characteristics across the grades of renal function were compared using the chi-square test, analysis of variance, or the Kruskal–Wallis test, as appropriate. The repeated measure linear mixed model was conducted to compare SBP observed in various grades over 7 days by treatment group, adjusting for baseline SBP. Associations between renal function and the efficacy and safety outcomes were estimated using logistic regression models: model 1 as univariate; model 2 with adjustment for minimal variables of age and sex; and model 3 as fully adjusted for variables of age, sex, time from stroke onset to randomization (hour), ethnicity, GCS score, NIHSS score, hypertension, coronary artery disease, diabetes mellitus, atrial fibrillation (AF), other heart disease, hypercholesterolemia, use of glucose-lowering treatment, smoking status, baseline SBP, baseline diastolic BP, premorbid mRS (0 or 1), premorbid use of aspirin, and randomized treatment (intensive vs. guideline-recommended BP-lowering treatment) as recorded at baseline. Logistic regression models were also used to compare the randomized treatment effect on the efficacy and safety outcomes across the three grades of renal function by adding an interaction term to the models. We also use eGFR as a continuous variable in logistic regression models to comprehensively estimate the relationship between eGFR and the outcomes of death, treatment effects, and sICH. Restricted cubic spline analysis was performed to examine the effect patterns, with 60 mL/min/1.73 m^2^ as the reference point. We conducted fractional polynomial analysis for the interaction effect between randomized treatment and continuous eGFR. Cause-specific mortality at 90 days was also assessed. Heterogeneity for the effect of renal function on mortality across three demography subgroups was assessed through tests for interaction, including age, sex, and ethnicity. As missing data for renal function accounted for less than 10% of the full dataset, we did not conduct imputation.

Data are reported with odds ratios (OR) and 95% confidence intervals (CI). Two-sided *p*-values were reported with *p <*0.05 considered as statistically significant. Data analyses were performed with SAS version 9.3 (SAS Institute): Cary, North Carolina, USA.R Foundation for Statistical Computing: Vienna, Austria.

## Results

Overall, 2,151 patients (mean age 66.9 years; 38.0% women) with available baseline renal function data were involved in this analysis, as outlined in **Supplementary Figure 1**. Baseline median eGFR was 86.9 (IQR 68.6–108.6) mL/min/1.73 m^2^, with 993 (46.2%), 822 (38.2%), and 336 (15.6%) patients identified as normal (G_1_), mild impairment (G_2_), and moderate-to-severe impairment (G_3_) of renal function. Demographic and clinical characteristics by baseline renal function category are outlined in **Table 1**. Patients with decreased eGFR were older, had more severe neurological impairment, and had greater frequency of hypertension, coronary artery disease, other heart disease, AF, diabetes mellitus, hypercholesterolemia, use of antiplatelet and glucose-lowering agents, and premorbid symptoms.

**Table 1:** Baseline characteristics by stages of renal function.

	eGFR category^a^, mL/min per 1.73 m^2^			*p-*value
	Stage G_1_ (*n* = 993)	Stage G_2_ (*n* = 822)	Stage G_3_ (*n* = 336)	
	≥90	60–89	<60	
Time from stroke onset to randomization (h)	3.4 (1.0)	3.3 (1.1)	3.4 (1.1)	0.017
Age (years)	62.9 (11.4)	68.5 (11.7)	74.5 (11.2)	<0.0001
≥80	60/993 (6.0)	136/822 (16.5)	117/336 (34.8)	<0.0001
Sex, female	335/993 (33.7)	323/822 (39.3)	160/336 (47.6)	<0.0001
Asian	877/993 (88.3)	547/822 (66.5)	169/336 (50.3)	<0.0001
Clinical features				
Systolic blood pressure, mmHg	164.6 (9.5)	165.8 (8.9)	165.7 (8.9)	0.009
Diastolic blood pressure, mmHg	92.4 (10.5)	90.6 (11.7)	87.4 (12.3)	<0.0001
Heart rate, bpm	78.1 (13.5)	80.0 (15.2)	81.3 (17.0)	0.001
GCS score^b^	15.0 (14.0, 15.0)	15.0 (14.0, 15.0)	15.0 (13.0, 15.0)	<0.0001
Severe (3–8)	35/993 (3.5)	37/822 (4.5)	12/336 (3.6)	0.532
NIHSS score^c^	7.0 (4.0, 11.0)	8.0 (5.0, 12.0)	9.0 (5.0, 14.0)	<0.0001
Severe, ≥14	163/993 (16.4)	169/822 (20.6)	93/336 (27.7)	<0.0001
Medical history				
Hypertension	667/993 (67.2)	599/822 (72.9)	272/336 (81.0)	<0.0001
Previous stroke	179/993 (18.0)	147/822 (17.9)	79/336 (23.5)	0.057
Coronary artery disease	106/993 (10.7)	118/822 (14.4)	73/336 (21.7)	<0.0001
Other heart disease	25/993 (2.5)	39/822 (4.7)	27/336 (8.0)	<0.0001
Atrial fibrillation	59/993 (5.9)	107/822 (13.0)	58/336 (17.3)	<0.0001
Diabetes mellitus	209/993 (21.0)	168/822 (20.4)	111/336 (33.0)	<0.0001
Hypercholesterolemia	60/993 (6.0)	116/822 (14.1)	66/336 (19.6)	<0.0001
Current smoker	249/993 (25.1)	142/822 (17.3)	46/334 (13.8)	<0.0001
Prestroke symptoms (mRS score 1)	97/992 (9.8)	122/822 (14.8)	86/336 (25.6)	<0.0001
Medications				
Antihypertensive agent(s)	70/993 (7.0)	52/820 (6.3)	34/335 (10.1)	0.073
Anticoagulation	8/993 (0.8)	13/822 (1.6)	7/336 (2.1)	0.135
Aspirin or other antiplatelet agents	122/993 (12.3)	157/822 (19.1)	97/336 (28.9)	<0.0001
Glucose lowering	111/993 (11.2)	101/822 (12.3)	82/336 (24.4)	<0.0001
*Final diagnosis at the time of hospital separation*				0.942
Non-stroke	10/980 (1.0)	8/816 (1.0)	4/332 (1.2)	
Presumed stroke pathology				<0.0001
Large artery occlusion due to significant atheroma	52/964 (5.4)	62/797 (7.8)	29/327 (8.9)	
Small vessel or perforating vessel lacunar disease	788/964 (81.7)	470/797 (59.0)	147/327 (45.0)	
Cardioembolism	55/964 (5.7)	141/797 (17.7)	84/327 (25.7)	
Dissection	2/964 (0.2)	4/797 (0.5)	1/327 (0.3)	
Other or uncertain etiology	67/964 (7.0)	120/797 (15.1)	66/327 (20.2)	

Data are shown as mean (SD), median (IQR), or *n*/*N* (%).

eGFR, estimated glomerular filtration rate; NIHSS, National Institutes of Health Stroke Scale; GCS, Glasgow Coma Scale; mRS, modified Rankin scale.

a Categories of estimated glomerular filtration rate: stage G_1_ (≥90 mL/min/1.73 m^2^), stage G_2_ (60–89 mL/min/1.73 m^2^), and stage G_3_ (<60 mL/min/1.73 m^2^).

b Scores on the GCS range from 15 (normal) to 3 (deep coma).

c Scores on the NIHSS range from 0 to 42, with higher scores indicating more severe neurological deficits.

**Supplementary Figure 2** depicts SBP management profiles in patients with different baseline renal functions, overall and by randomized treatment groups. Mean SBP within 7 days was higher in patients with moderate-to-severe impairment of eGFR (G_3_) than in the other two groups (*p* < 0.0001), in both BP-lowering groups.

**Figure 1:**
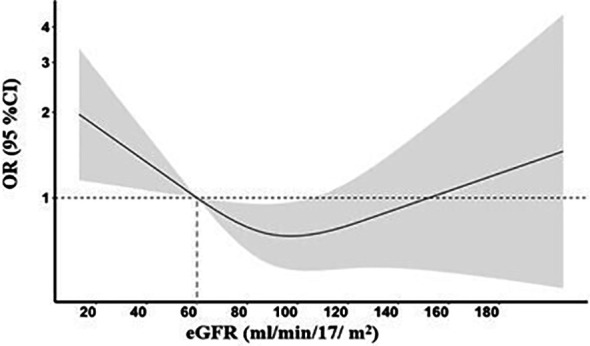
Risk of death across the baseline estimated glomerular filtration rate (eGFR). A restricted cubic spline curve with three knots (53.5, 86.9, and 130.2) illustrating the association of the baseline eGFR with odds ratios (solid line) and 95% confidence intervals (gray area). The model was adjusted for age, sex, time from stroke onset to randomization (hour), ethnicity, GCS score, NIHSS score, hypertension, coronary artery disease, diabetes mellitus, atrial fibrillation, other heart disease, hypercholesterolemia, glucose-lowering treatment, smoker, baseline systolic BP, baseline diastolic BP, premorbid mRS (0 or 1), premorbid use of aspirin, and randomized treatment (intensive vs. guideline-recommended BP-lowering treatment).

There was no statistically significant association of impaired renal function with either death or major disability [mRS scores 3 to 6; adjusted odds ratio (aOR) 1.06, 95% confidence interval (CI) 0.78–1.45; *p =* 0.940 for trend] or major disability (mRS scores 3 to 5, aOR 1.01, 95% CI 0.72–1.43; *p* = 0.708 for trend) at 90 days. However, patients with stage G_3_ had nearly two-fold greater odds of death compared to those with stage G_1_ (aOR 1.77, 95% CI 1.05–2.99; *p* = 0.031 for trend) within 90 days (**Table 2**). There is no multicollinearity in covariates in the above models. A non-linear association between baseline eGFR as a continuous variable and death after adjustment for other variables is shown in **Figure 1**, and the OR of death was statistically significantly increased where the eGFR was <60 mL/min/1.73 m^2^.

**Table 2:** Efficacy outcomes at 90 days by stages of renal function.

Outcome	*n*/*N* (%)	Unadjusted model		Model 1^a^		Model 2^b^	
		OR (95% CI)	*p* trend	aOR (95% CI)	*p* trend	aOR (95% CI)	*p* trend
Major disability (mRS scores 3–5)							
Stage G_1_ ^c^	231/935 (24.7)	1	0.0003	1	0.170	1	0.708
Stage G_2_	195/737 (26.5)	1.10 (0.88–1.37)		0.94 (0.75–1.18)		0.83 (0.64–1.07)	
Stage G_3_	105/281 (37.4)	1.81 (1.37–2.41)		1.34 (0.99–1.81)		1.01 (0.72–1.43)	
Death at day 90 (mRS score 6)							
Stage G_1_	55/990 (5.6)	1	<0.0001	1	0.0003	1	0.031
Stage G_2_	78/815 (9.6)	1.91 (1.32–2.74)		1.55 (1.07–2.26)		1.47 (0.94–2.29)	
Stage G_3_	53/334 (15.9)	3.34 (2.22–5.02)		2.23 (1.44–3.45)		1.77 (1.05–2.99)	
Death or major disability (mRS scores 3–6)							
Stage G_1_	286/990 (28.9)	1	<0.0001	1	0.079	1	0.525
Stage G_2_	273/815 (33.5)	1.25 (1.03–1.53)		1.05 (0.85–1.30)		0.92 (0.72–1.16)	
Stage G_3_	158/334 (47.3)	2.22 (1.72–2.87)		1.57 (1.19–2.06)		1.18 (0.85–1.62)	

mRS, modified Rankin scale; OR, odds ratio; aOR, adjusted odds ratio; CI, confidence interval.

a Model 1: adjusted analysis for minimization variables including baseline variables: age and sex.

b Model 2: based on model 1, additional adjustment for time from stroke onset to randomization (hour), ethnicity, GCS score, NIHSS score, hypertension, coronary artery disease, diabetes mellitus, atrial fibrillation, other heart disease, hypercholesterolemia, glucose-lowering treatment, smoker, baseline systolic BP, baseline diastolic BP, premorbid mRS (0 or 1), premorbid use of aspirin, and randomized treatment (intensive vs. guideline-recommended BP-lowering treatment).

c Categories of estimated glomerular filtration rate: stage G_1_ (≥90 mL/min/1.73 m^2^), stage G_2_ (60–89 mL/min/1.73 m^2^), and stage G_3_ (<60 mL/min/1.73 m^2^).

**Figure 2:**
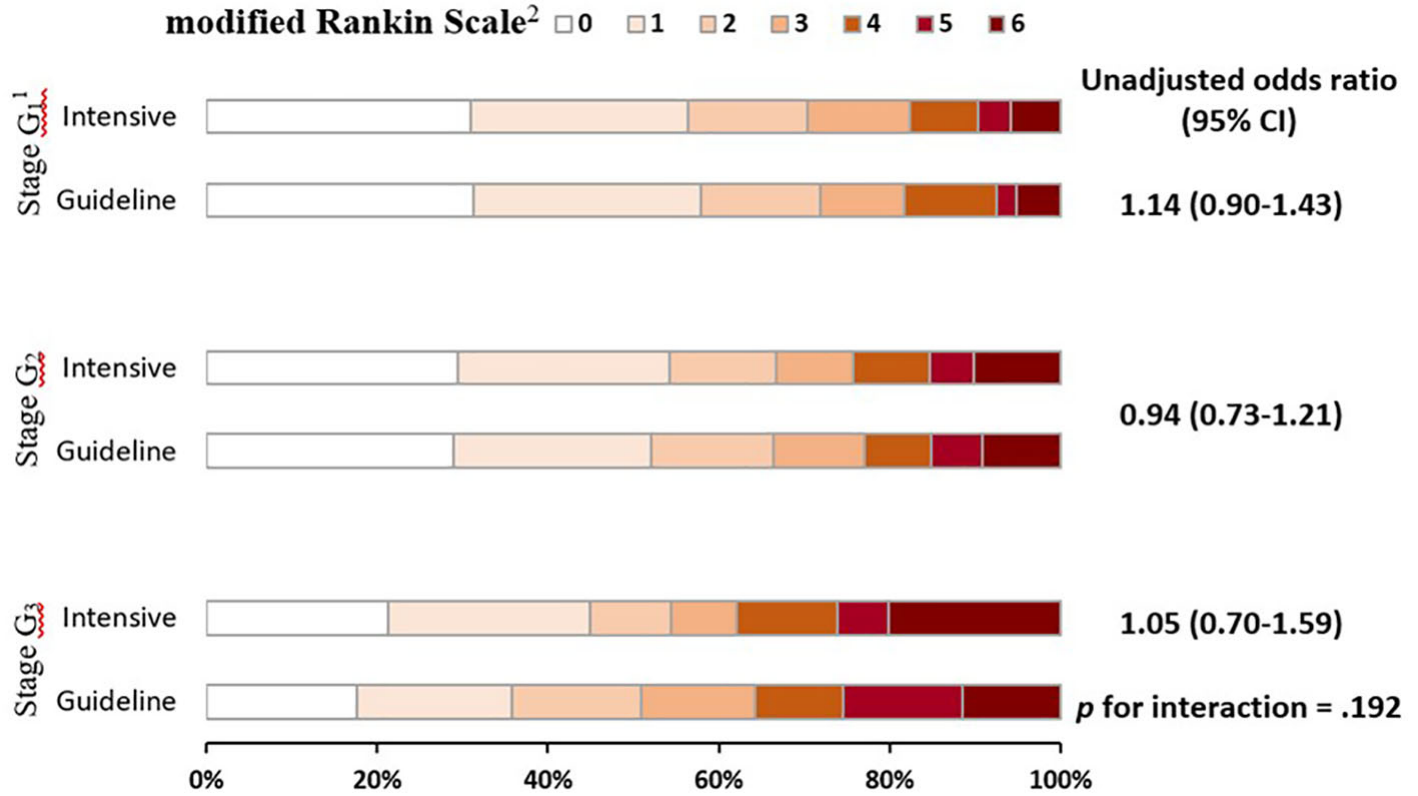
Global functional outcome at 90 days in patients by stages of renal function and randomized treatment. Raw distribution of scores is shown by renal failure stages. ^1^Scores on the modified Rankin scale range from 0 to 6, with 0 indicating no symptoms, 1 symptoms without clinically significant disability, 2 slight disability, 3 moderate disability, 4 moderately severe disability, 5 severe disability, and 6 death. ^2^Categories of estimated glomerular filtration rate: stage G_1_ (≥90 mL/min/1.73 m^2^), stage G_2_ (60–89 mL/min/1.73 m^2^), and stage G_3_ (<60 mL/min/1.73 m^2^).

There was no statistically significant interaction between stages of renal function and randomized treatment effect on clinical outcomes, including for an ordinal shift in mRS scores (*p* for interaction = 0.192) (**Figure 2**) and others (**Table 3**). In fractional polynomial analysis, no significant interaction effect on death was shown between continuous eGFR value and randomized treatment. Patients with decreased eGFR at baseline did not increase the risk of death significantly in the intensive BP-lowering group (*p* for interaction = 0.853) (**Supplementary Figure 3**).

**Table 3:** Categorical primary outcome at 90 days by stages of renal function and randomized treatment.

Outcome	Randomized treatment		aOR (95% CI)^a^	*p* for interaction
	Guideline-recommended BP-lowering group	Intensive BP-lowering group		
Major disability (mRS scores 3–5)				
Stage G_1_ ^b^	114/472 (24.2)	117/463 (25.3)	1.21 (0.87–1.67)	0.120
Stage G_2_	105/389 (27.0)	90/348 (25.9)	0.87 (0.61–1.26)	
Stage G_3_	62/146 (42.47)	43/135 (31.85)	0.69 (0.39–1.21)	
Death or major disability (mRS scores 3–6)				
Stage G_1_	140/498 (28.1)	146/492 (29.7)	1.20 (0.88–1.64)	0.410
Stage G_2_	144/468 (33.6)	129/387 (33.3)	0.96 (0.69–1.34)	
Stage G_3_	81/165 (49.1)	77/169 (45.6)	0.97 (0.59–1.60)	
Death (mRS score 6)				
Stage G_1_	26/498 (5.2)	29/492 (5.9)	1.22 (0.59–2.55)	0.545
Stage G_2_	39/468 (9.1)	39/387 (10.1)	1.35 (0.78–2.31)	
Stage G_3_	19/165 (11.5)	34/169 (20.1)	3.29 (1.51–7.17)	

Frequency data are *n*/*N* (%).

a Estimates from a logistic regression model with adjustment for age, sex, time from stroke onset to randomization (hour), ethnicity, GCS score, NIHSS score, hypertension, coronary artery disease, diabetes mellitus, atrial fibrillation (AF), other heart disease, hypercholesterolemia, glucose-lowering treatment, smoker, baseline systolic BP, baseline diastolic BP, premorbid mRS (0 or 1), premorbid use of aspirin, and randomized treatment (intensive vs. guideline-recommended BP-lowering treatment).

b Categories of estimated glomerular filtration rate: stage G_1_ (≥90 mL/min/1.73 m^2^), stage G_2_ (60–89 mL/min/1.73 m^2^), and stage G_3_ (<60 mL/min/1.73 m^2^).

**Figure 3:**
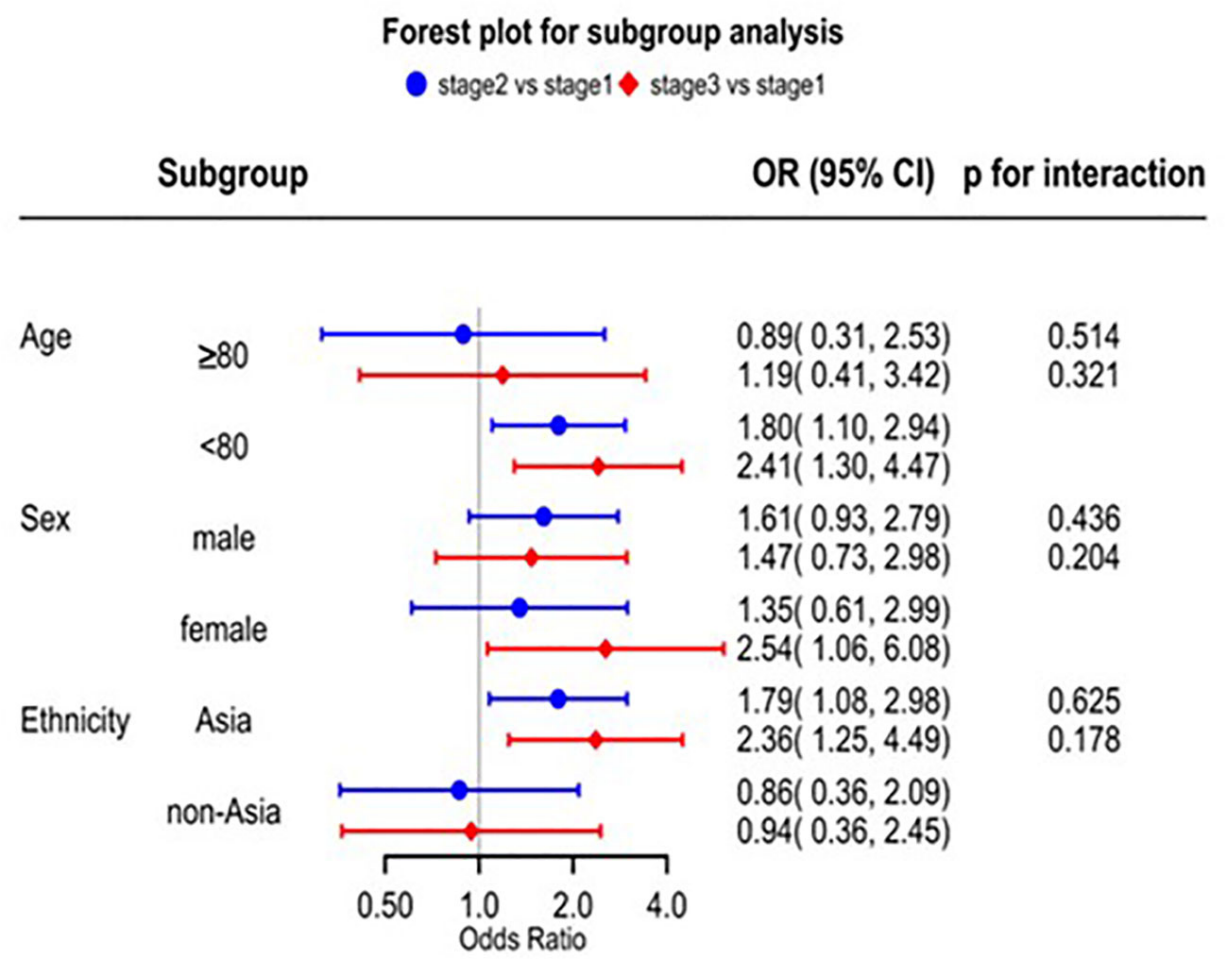
Risk of death in subgroups.

Patients with decreased eGFR had a higher risk of sICH according to the SITS-MOST and other definitions (**Supplementary Table 1**). A series of splines display the non-linear relationship between baseline continuous eGFR (eGFR 60 mL/min/1.73 m^2^ as the reference) and sICH. For eGFR <60 mL/min/1.73 m^2^, the OR for sICH increased with decreased baseline eGFR (**Supplementary Figure 4**). Patients with decreased eGFR had a higher risk of death caused by the direct effects of acute ischemic stroke and non-vascular reasons (**Supplementary Table 2**). There was no significant interaction between renal impairment and the three subgroups on mortality (**Figure 3**). However, we found that the risk of mortality was significantly higher in the Asian subgroup than in the non-Asian subgroup. In the Asian group, stage G_2_ had nearly two-fold greater odds of death compared to those with stage G_1_ (aOR 1.79, 95% CI 1.08–2.98) and stage G_3_ (more than two-fold) (aOR 2.36, 95% CI 1.25–4.49) (**Figure 3**).

## Discussion

In this *post-hoc* analysis of the ENCHANTED BP arm, moderate-to-severe impairment of eGFR was adversely associated with mortality and risk of sICH but not with functional outcome in thrombolyzed patients with AIS. We also found that patients with moderate-to-severe renal impairment maintained a higher SBP compared to those with normal or mild renal impairment, regardless of the randomized treatment allocation. There was no evidence of heterogeneity in the effect of intensive BP-lowering treatment on death according to different renal function groups.

Renal dysfunction was proven to increase mortality in critical illnesses including AIS (8, 17–20). Our study further adds to this knowledge by defining the association specifically in thrombolyzed AIS and is consistent with an earlier analysis of the ENCHANTED alteplase-dose arm and other studies (8, 21). The relationship between eGFR and mortality was non-linear, and the odds of death were statistically significantly higher in patients whose baseline eGFR was less than 60 mL/min/1.73 m^2^. Potential explanations are that patients with renal impairment exhibit other organ dysfunction, which increases the risk of death (22). We did not find any statistically significant association between renal impairment and disability, as observed in other studies (23, 24), but ICH was higher in those patients with pre-existing renal impairment, which may have potentiated effects on the coagulation system and morphologically changed blood vessel walls through interactions between the endothelium and platelets (25, 26). Patients with renal impairment have varying degrees of systematic inflammation, which also influences the risk of bleeding (27), while dialysis treatment is known to produce a hypocoagulable state and exacerbate the likelihood of ICH (28). Thus, weighing up the potential risks and benefits of thrombolysis treatment in patients with chronic kidney disease can be challenging.

In our study, patients with severe renal impairment had more hypertension and higher baseline BP and maintained higher BP over the subsequent 7 days compared to those with normal or mildly reduced renal function, which potentially reflects the complex interaction between renal function and response to BP-lowering treatment in AIS, as shown elsewhere (12). Renal impairment is a common cause of hypertension, and there was a higher frequency of history of hypertension at baseline in the G_3_ compared to the other two groups (29). The higher mean SBP achieved within 6 h raises the possibility that renal impairment contributes to the therapeutic resistance to antihypertensive agents through such mechanisms as increased sympathetic activity, alterations in the renin–angiotensin–aldosterone system, vascular aging and atherosclerosis, and sodium dysregulation from reduced functioning nephrons and lowered glomerular filtration rate (29, 30). Of course, clinicians may have also altered the intensity of their BP-lowering treatment from concerns about inducing renal impairment in such high-risk patients (31).

There was no heterogeneity in the BP-lowering treatment effect on death across different stages of renal impairment. Although the rate of death appeared higher in the intensive BP-lowering group with decreased eGFR, this finding lacked precision due to small numbers. This might be a reminder for clinicians to be cautious about the use of intensive BP-lowering treatment in patients with known renal impairment, but more data are needed to confirm this finding. Subgroup analysis results of ethnicity found that renal impairment might lead to a higher risk of mortality in Asians. As there was no heterogeneity, more studies are needed in the future to confirm it.

The strengths of our study include the prospective design with high rates of follow-up, treatment compliance, and rigorous assessment of outcomes in a relatively large sample of patients with a broad range of characteristics. Our analyses included the use of restricted cubic spline models and various sensitive analyses to describe the relationship between eGFR and outcomes. However, our study has limitations that include the dataset pertaining to a clinical trial population where most patients were of Asian ethnicity where the CKD-EPI equation, which was derived from North American and European populations, may have measurement bias. Selection bias may have also led to the exclusion of patients with severe AIS where the risks of intensive BP-lowering treatment were perceived to be high, thus further limiting the generalizability of our findings. Furthermore, the use of baseline/admission eGFR as the exposure marker may be misleading as serum creatinine is prone to elevation by acute illness (32), and the definitions for renal impairment normally require persistence of eGFR <60 mL/min per 1.73 m^2^ for at least 3 months in the absence of a reversible condition (33). Finally, the ENCHANTED trial did not demonstrate an improvement in functional outcomes, primarily due to the minimal difference in mean SBP between the intensive BP-lowering group and the control group. Consequently, a *post-hoc* analysis might inherently possess limitations in addressing the research question of this article.

## Conclusion

In summary, our analysis of the ENCHANTED BP arm clinical trial dataset has shown that renal impairment predicts higher mortality in thrombolyzed AIS patients. Patients with reduced eGFR might also have a higher risk of sICH. Uncertainty persists as to whether intensive BP-lowering treatment confers benefits over guideline-recommended treatment in AIS patients with renal impairment.

## Data Availability

The raw data supporting the conclusions of this article will be made available by the authors, without undue reservation.
