# Supplementary material for: Influence of renal function on blood pressure control and outcome in thrombolyzed patients after acute ischemic stroke: post-hoc analysis of the ENCHANTED trial
Source: Front Endocrinol (Lausanne). 2024 Dec 9;15:1341902. doi: 10.3389/fendo.2024.1341902 (PMC11663659; doi:10.3389/fendo.2024.1341902)
Supplement: Supplementary file 1 [file DataSheet1.docx]

**SUPPLEMENTAL MATERIALS**

**Supplemental Tables 1-3**

**Supplemental Figures 1-4**

**Supplement Table 1. Key secondary outcome of symptomatic intracerebral hemorrhage across all definitions by stages of renal function**

|  | **eGFR Category**^1^**, ml/min per 1.73 m^2^** | | | ***p* trend** |
| --- | --- | --- | --- | --- |
|  | **Stage G_1_ (n=993)** | **Stage G_2_ (n=822)** | **Stage G_3_ (n=336)** |  |
| SITS-MOST | 12/993 (1.2) | 15/882 (1.8) | 7/336 (2.1) | .201 |
| NINDS | 54/993 (5.4) | 69/882 (8.4) | 26/336 (7.7) | .041 |
| ECASS2 | 35/993 (3.5) | 46/882 (5.6) | 19/336 (5.7) | .041 |
| ECASS3 | 16/993 (1.6) | 22/882 (2.7) | 11/336 (3.3) | .049 |
| IST-3 | 18/993 (1.8) | 28/882 (3.4) | 13/336 (3.9) | .019 |
| Clinician reported any Intracranial haemorrhage | 52/993 (5.2) | 73/882 (8.9) | 30/336 (8.9) | .004 |
| Fatal ICH | 7/993 (0.7) | 5/882 (0.6) | 5/336 (1.5) | .287 |
| Adjudicated any ICH | 119/993 (12.0) | 137/882 (16.7) | 56/336 (16.7) | .007 |
| Any Intracranial haemorrhage | 137/993 (13.8) | 156/882 (19.0) | 63/336 (18.8) | .006 |

Data are shown as n/N (%).

^1^ Categories of estimated glomerular filtration rate: Stage G_1_ (≥90 ml/min/1.73m^2^), Stage G_2_ (60-89 ml/min/1.73m^2^), Stage G_3_ (<60 ml/min/1.73m^2^).

Abbreviations: SITS-MOST=Safe Implementation of Thrombolysis in Stroke Monitoring Study. NINDS=National Institute of Neurological Disorders and Stroke. ECASS=European Co-operative Acute Stroke Study. IST=International Stroke Trial, ICH: intracerebral hemorrhage.

**Supplement Table 2: Causes of death by stages of renal function**

| **Outcome** | **eGFR Category**^1^**, ml/min per 1.73 m^2^** | | | ***p* value** |
| --- | --- | --- | --- | --- |
|  | **Stage G_1_ (n=993)** | **Stage G_2_ (n=822)** | **Stage G_3_ (n=336)** |  |
| Direct effects of the acute ischemic stroke | 28/993 (2.8%) | 30/822 (3.6%) | 22/336 (6.5%) | .008 |
| Acute intracerebral hemorrhage | 11/993 (1.1%) | 12/822 (1.5%) | 9/336 (2.7%) | .120 |
| Recurrent stroke |  |  |  |  |
| Acute intracerebral hemorrhage | 0/993(0.0%) | 0/822(0.0%) | 0/336(0.0%) | - |
| Acute ischemic stroke | 2/993 (0.2%) | 2/822 (0.2%) | 2/336 (0.6%) | .482 |
| Undifferentiated stroke | 1/993 (0.1%) | 0/822(0.0%) | 0/336(0.0%) | .558 |
| Acute coronary event | 3/993 (0.3%) | 2/822 (0.2%) | 2/336 (0.6%) | .625 |
| Other vascular | 2/993 (0.2%) | 8/822 (1.0%) | 1/336 (0.3%) | .060 |
| Non-vascular | 8/993 (0.8%) | 25/822 (3.0%) | 17/336 (5.1%) | <.0001 |

^1^ Categories of estimated glomerular filtration rate: Stage G_1_ (≥90 ml/min/1.73m^2^), Stage G_2_ (60-89 ml/min/1.73m^2^), Stage G_3_ (<60 ml/min/1.73m^2^).

| **Supplement Table 3: Symptomatic intracerebral hemorrhage at 90 days by stages of renal function and randomized treatment** | | | | |
| --- | --- | --- | --- | --- |
| **Outcome** | **Randomized treatment** | | **aOR (95% CI)**^1^ | ***p* for interaction** |
|  | **Guideline recommended BP lowering group** | **Intensive BP lowering group** |  |  |
| SITS-MOST | 22/1115 (1.97) | 14/1081 (1.30) | 0.77 (0.34-1.73) | 0.474 |
| NINDS | 84/1115 (7.53) | 70/1081 (6.48) | 0.87 (0.61-1.25) | 0.178 |
| ECASS2 | 57/1115 (5.11) | 46/1081 (4.62) | 0.89 (0.57-1.39) | 0.283 |
| ECASS3 | 30/1115 (2.69) | 21/1081 (1.94) | 0.82 (0.43-1.57) | 0.528 |
| IST-3 | 37/1115 (3.32) | 24/1081 (2.22) | 0.75 (0.42-1.36) | 0.384 |
| Clinician reported any Intracranial haemorrhage | 100/1115 (8.97) | 59/1081 (5.46) | 1.19 (0.81-1.75) | 0.540 |
| Fatal ICH | 14/1115 (1.26) | 5/1081 (0.46) | 0.41 (0.07-2.29) | 0.987 |
| Adjudicated any ICH | 180/1115 (16.14) | 143/1081 (13.23) | 0.97 (0.59-1.60) | 0.763 |
| Any Intracranial haemorrhage | 209/1115 (18.74) | 160/1081 (14.80) | 0.73 (0.57-0.93) | 0.730 |

^1^Estimates from a logistic regression model with adjustment for age, sex, time from stroke onset to randomisation (hour), ethnicity, GCS score, NIHSS score, hypertension, coronary artery disease, diabetes mellitus, atrial fibrillation (AF), other heart disease, hypercholesterolemia, glucose lowering treatment, smoker, baseline systolic BP, baseline diastolic BP, pre-morbid mRS (0 or 1), pre-morbid use of aspirin, and randomized treatment (intensive vs. guideline-recommended BP lowering).

**Supplemental Figure 1: Flow Chart**

**Supplemental Figure 2. Mean systolic blood pressure (SBP) profile in 7 days from randomization grouped by treatment and stages of renal function**

1. **All**


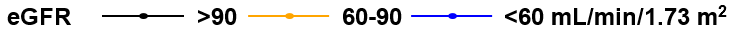

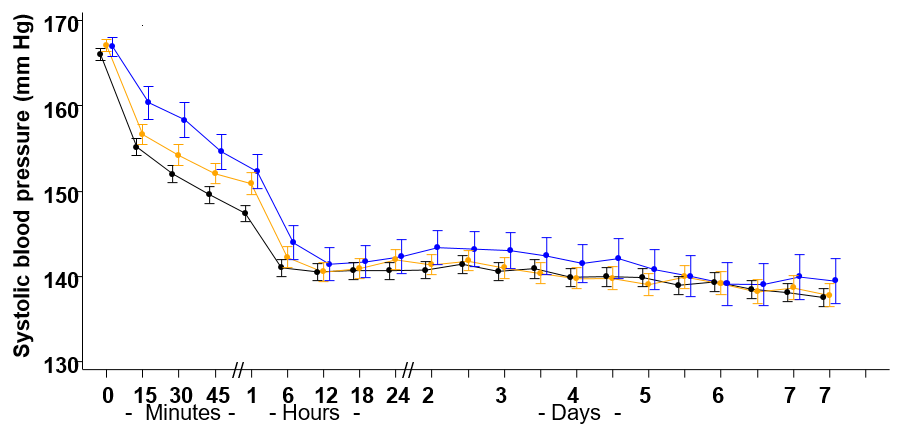


*p* < .0001

1. **Guideline-recommended group**


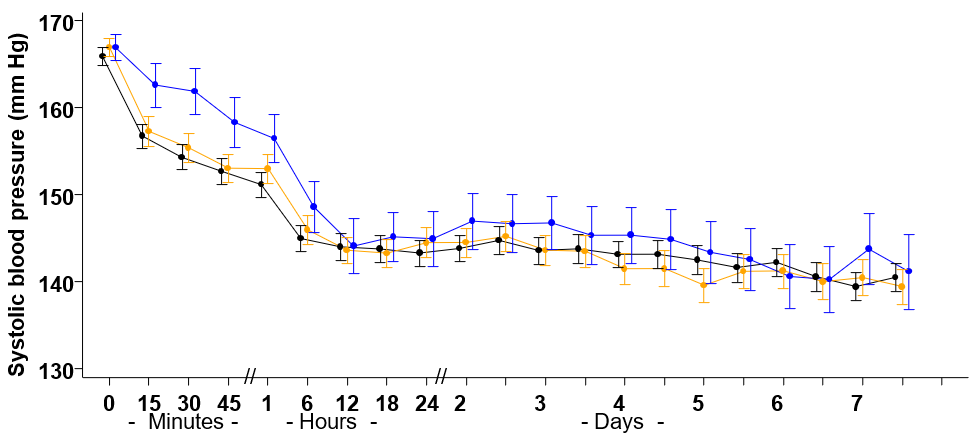


*p* < .0001

1. **Intensive group**


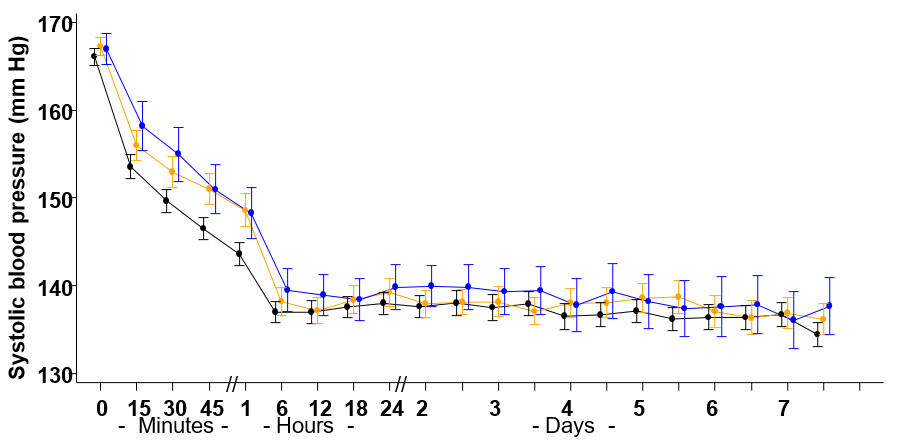


*p* < .0001

Values are shown for the level of systolic blood pressure by renal impairment stages of renal function in all patients (A), in guideline-recommended blood pressure control group (B) and in intensive blood pressure lowering group (C). Recordings were at 15-minute intervals in the ﬁrst hour after randomisation (time 0), 6-hourly until 24 hours, and twice daily until Day 7. The differences in variables between renal function stages were assessed using a repeated measure linear mixed model, adjusting for baseline SBP.

**Supplemental Figure 3: Risk of death comparing two treatment groups by baseline eGFR (ml/min/1.73 m^2^)**


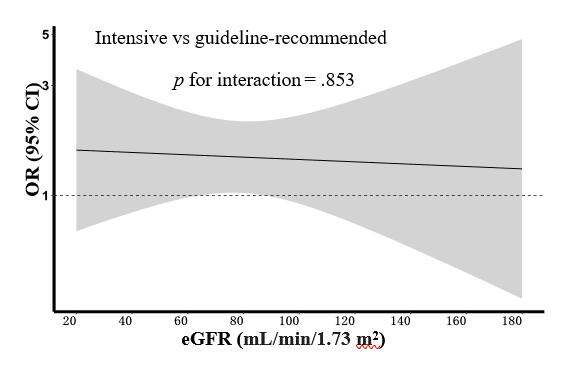


The multivariable fractional polynomial interaction plot shows the adjusted odds ratios (solid line) of death, comparing intensive vs guideline-recommended BP-lowering treatment across continuous values of baseline eGFR. The grey area shows 95% confidence intervals. Models were adjusted for age, sex, time from stroke onset to randomisation (hour), ethnicity, GCS score, NIHSS score, hypertension, coronary artery disease, diabetes mellitus, atrial fibrillation (AF), other heart disease, hypercholesterolemia, glucose lowering treatment, smoker, baseline systolic BP, baseline diastolic BP, pre-morbid mRS (0 or 1), pre-morbid use of aspirin.

**Supplemental Figure 4. Risk of symptomatic intracerebral hemorrhage across the baseline eGFR) (ml/min/1.73 m^2^)**

Abbreviations:
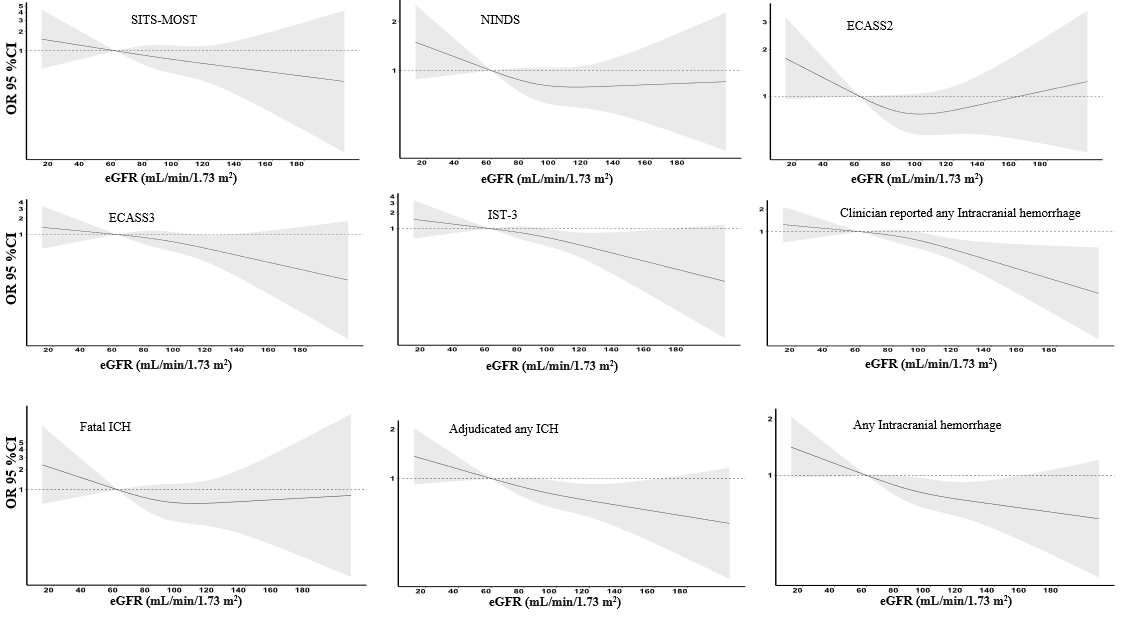
 SITS-MOST = Safe Implementation of Thrombolysis in Stroke Monitoring Study. NINDS = National Institute of Neurological Disorders and Stroke.

ECASS = European Co-operative Acute Stroke Study. IST = International Stroke Trial. ICH=intracerebral hemorrhage.
